# Molecular Characterization of the Dominant-Negative Role of Cancer-Associated PTEN: Sometimes, Null is Better

**DOI:** 10.3389/fonc.2014.00276

**Published:** 2014-10-10

**Authors:** Saverio Marchi, Paolo Pinton

**Affiliations:** ^1^Laboratory for Technologies of Advanced Therapies (LTTA), Section of Pathology, Oncology and Experimental Biology, Department of Morphology, Surgery and Experimental Medicine, University of Ferrara, Ferrara, Italy

A number of observations have radically expanded our knowledge regarding the signaling pathways that regulate the tumor suppressor PTEN. These findings are derived from the molecular characterization of cancer-associated dominant-negative PTEN mutants, as well as the identification of alternative forms and intracellular localizations of PTEN.

The lipid and protein phosphatase PTEN (Phosphatase and tensin homolog located on chromosome 10) is one of the most common tumor suppressors that is lost or mutated in several human cancers. Different mechanisms contribute to PTEN loss of function, which include post-translational modifications, interaction with other proteins, missense and truncation mutations, as well as gene deletions. The role of PTEN as an oncosuppressor has mainly been characterized through the study of different *PTEN*-null tumor cell lines or transgenic mice engineered to carry one wild-type (WT) copy and one null allele of *PTEN* (*PTEN*^+/−^). However, a paper published in 1998 by the group of Eng and co-workers reported that *PTEN* loss-of-function mutations might not contribute to tumorigenesis to the same extent as the genetic loss of *PTEN*. This study was conducted on patients affected by Cowden disease, a PTEN hamartoma tumor syndrome, and revealed that patients with missense PTEN mutations in the phosphatase domain developed higher numbers of lesions compared to patients with truncating mutations (1). The mechanistic and molecular role of PTEN mutations in cancer has been recently defined in a paper published in *Cell* (2). Papa et al. showed that different PTEN mutants, previously identified in tumors (i.e., PTEN C124S, PTEN G129E, and PTEN R130G), form heterodimeric structures with the WT enzyme, thereby inhibiting PTEN activity in a dominant-negative manner and leading to aberrant Akt activation (2). Moreover, it has been observed that dephosphorylation of the PTEN tail not only promotes an open conformation of the enzyme but also favors the formation of dimers or oligomers (2). The heterozygous expression of cancer-associated PTEN mutations allows the generation of catalytically inactive heterodimers. Consequently, increased PIP3 levels induce Akt hyperactivation and augment tumor progression. Thus, a neoplasm characterized by PTEN mutations is more unfavorable than a PTEN-null cancer context. Interestingly, this now reveals that the malignant activity of mutant PTENs is analogous to that of mutant p53: p53, a tumor suppressor also frequently mutated in cancers, normally functions in a tetrameric conformation; moreover, p53 mutants can similarly act as dominant-negative inhibitors of residual WT protein (3).

The ability of mutant PTEN to heterodimerize and inhibit the WT enzymatic activity is one of the recent findings that have radically expanded our knowledge on PTEN activity and functions. Firstly, the identification of an alternative variant of PTEN, named PTEN-Long, containing an extra 173 aminoacidic domain at its N-terminus, followed by the canonical PTEN sequence (4). The resulting 576-aminoacids protein is less abundant than the short isoform; it has been founded to be mutated in tumor samples and can be secreted to enter other cells (4, 5).

Furthermore, it has been shown as PTENα, a 70-kDa PTEN variant that most likely corresponds to PTEN-Long (5), controls mitochondrial metabolism and the energy status of the cell (6). PTENα can interact with canonical PTEN, forming heterodimers that regulate mitochondrial bioenergetics (6). Interestingly, transgenic mouse lines with PTEN expression displayed increased mitochondrial oxidative phosphorylation and ATP production (7). Unlike canonical PTEN, which plays a well-defined role in signaling at the plasma membrane and nucleus, PTENα localizes mainly to the cytosol and mitochondria (6). In line with this, alternative subcellular localizations of PTEN, such as to the nucleoli (8) and mitochondria-associated endoplasmic reticulum membranes (MAM) (9), have been recently described. Our group demonstrated that PTEN interacts with the inositol-1,4,5-trisphosphate receptor (IP3R) at endoplasmic reticulum (ER) to counteract Akt activity, consequently favoring calcium (Ca^2+^) transfer into the mitochondria and the initiation of apoptosis (9, 10). Accordingly, we and other labs proposed that Akt kinase might potentiate its anti-apoptotic activity through IP3R phosphorylation and the reduction of Ca^2+^ release from the ER (11–14). Thus, PTEN acts at multiple subcellular levels, regulating PI3K signaling through its phosphatase activity.

Although the ER-targeted C124S and G129E PTEN chimeras still maintain their ability to suppress Akt activation (9), there is currently no evidence that cancer-associated PTEN mutants operate at specific subcellular compartments. Nevertheless, upon starvation/serum stimulation, C124S and G129E mutants translocate more rapidly to the plasma membrane than the WT form (2).

The very recent characterization of a bioluminescence resonance energy transfer (BRET)-based biosensor, which is capable of detecting signal-dependent PTEN conformational changes in live cells (15), may represent a valuable tool for elucidating PTEN activity and functions.

## Conflict of Interest Statement

The authors declare that the research was conducted in the absence of any commercial or financial relationships that could be construed as a potential conflict of interest.
